# Transcriptome Sequencing and Analysis of Wild Amur Ide (*Leuciscus waleckii*) Inhabiting an Extreme Alkaline-Saline Lake Reveals Insights into Stress Adaptation

**DOI:** 10.1371/journal.pone.0059703

**Published:** 2013-04-01

**Authors:** Jian Xu, Peifeng Ji, Baosen Wang, Lan Zhao, Jian Wang, Zixia Zhao, Yan Zhang, Jiongtang Li, Peng Xu, Xiaowen Sun

**Affiliations:** 1 Centre for Applied Aquatic Genomics, Chinese Academy of Fishery Sciences, Beijing, China; 2 College of Life Sciences, Tianjin Normal University, Tianjin, China; Auburn University, United States of America

## Abstract

**Background:**

Amur ide (*Leuciscus waleckii*) is an economically and ecologically important species in Northern Asia. The Dali Nor population inhabiting Dali Nor Lake, a typical saline-alkaline lake in Inner Mongolia, is well-known for its adaptation to extremely high alkalinity. Genome information is needed for conservation and aquaculture purposes, as well as to gain further understanding into the genetics of stress tolerance. The objective of the study is to sequence the transcriptome and obtain a well-assembled transcriptome of Amur ide.

**Results:**

The transcriptome of Amur ide was sequenced using the Illumina platform and assembled into 53,632 cDNA contigs, with an average length of 647 bp and a N50 length of 1,094 bp. A total of 19,338 unique proteins were identified, and gene ontology and KEGG (Kyoto Encyclopedia of Genes and Genomes) analyses classified all contigs into functional categories. Open Reading Frames (ORFs) were detected from 34,888 (65.1%) of contigs with an average length of 577 bp, while 9,638 full-length cDNAs were identified. Comparative analyses revealed that 31,790 (59.3%) contigs have a significant similarity to zebrafish proteins, and 27,096 (50.5%), 27,524 (51.3%) and 27,996 (52.2%) to teraodon, medaka and three-spined stickleback proteins, respectively. A total of 10,395 microsatellites and 34,299 SNPs were identified and classified. A dN/dS analysis on unigenes was performed, which identified that 61 of the genes were under strong positive selection. Most of the genes are associated with stress adaptation and immunity, suggesting that the extreme alkaline-saline environment resulted in fast evolution of certain genes.

**Conclusions:**

The transcriptome of Amur ide had been deeply sequenced, assembled and characterized, providing a valuable resource for a better understanding of the Amur ide genome. The transcriptome data will facilitate future functional studies on the Amur ide genome, as well as provide insight into potential mechanisms for adaptation to an extreme alkaline-saline environment.

## Introduction

Amur ide (*Leuciscus waleckii*) is a species of cyprinid fish, inhabiting the Amur River basin in Russia, Mongolia, China and Korea (www.fishbase.org). There are many local populations inhibiting lakes and watersheds in Northern Asia. One of the most renowned populations is the Dali Nor population which inhabits Dali Nor Lake, Inner Mongolia (E 116°25′–116°45′,N43°13′–43°23′ ). Dali Nor Lake is a typical saline-alkaline lake with high concentrations of carbonate salts. It is located in a basin of the eastern Inner Mongolia Plateau where outflow is completely prevented (an endothecia basin), and the evaporation is greater than precipitation and inflow. Thus, the lake has been shrinking consistently since the early Holocene (11,500–7,600 cal yr BP), and the alkalinity and salinity are increasing steadily [1]. Currently pH values range from 8.25 to 9.6, the alkali content (ALK) is over 50 mg/L, and salinity is ∼6 ‰. There are only two fish species, Amur ide and crucian carp, that have adapted to the extreme conditions and are able to survive in this harsh environment. Amur ide is economically important to the local Mongolian people who have lived around Dali Nor Lake for generations. Selective breeding program on Amur ide has been recently initiated on both growth and alkaline tolerance. The strain with better performance would be important for the rural region with large saline-alkaline open water in northern China. Besides, it is ecologically important fish to migrating birds that stop over at Dali Nor Lake and feed on Amur ide during their journeys from Siberia to the south [2].

In spite of its economic and ecological importance, the mechanism of Amur ide’s tolerance of the high salinity and alkalinity of Dali Nor Lake is still unclear. Very limited physiological and genetic studies have been performed, and information on its genetic resources has been only minimally developed. To date, only a few genetic markers have been developed [3]. The genetic diversity and population structure have been investigated by using microsatellite markers of Amur ide and grass carp. This study revealed that the Dali Nor population of Amur ide is genetically distinct from the Ussuri River population of Amur ide, suggesting that the Dali Nor population was isolated geologically a long time ago [4]. The mitochondrial genome of Amur ide from Dali Nor Lake has been completely sequenced and annotated, providing some basic molecular tools for ecological and genetic studies [5]. Amur ide has been recently developed as a potential aquaculture species in the widely distributed saline and alkaline waters of northern China because of its high tolerance to increased salinity and alkalinity. To better understand the physiological and genetic basis of its salinity tolerance, to explore adaptive evolution in its genome, and to support prospective genetic breeding and stock improvement, more knowledge about genetics and genome resources are needed.

EST (Expressed sequence tag) sequencing has been considered an efficient approach for genomic study and functional gene identification, especially for those species without a genome reference. In the past decade, tens of thousands ESTs have been developed for several important aquaculture species using traditional Sanger’s methods (http://www.ncbi.nlm.nih.gov/dbEST/dbEST_summary.html), including catfish (500,000 ESTs) [6], Atlantic salmon (498,212 ESTs), rainbow trout (287,967 ESTs), as well as aquatic parasite species such as *Ichthyophthirius multifiliis* (33,516 ESTs) [7]. These EST resources allow efficient gene discovery and transcriptome profiling in these species [8]–[12], as well as genetic marker development [13]. In the past half-decade, high-throughput next-generation sequencing technologies have been developed and successfully exploited to obtain a vast amount of transcriptome sequences at a lower cost, providing scientists the ability to collect sufficient genetic and genome resources for the many different species [14]–[16].

In the present study, we sequenced the transcriptome of Amur ide with the Illumina sequencing platform. The transcriptome sequences were assembled into contigs. Function annotation and gene ontology analyses were performed, and a large amount of microsatellite and SNP loci were identified. Synonymous and non-synonymous sites were analyzed to identify those genes that may be under strong positive selection in extreme environment. It is the first high throughput data for the genus *Leuciscus*, which provides a valuable resource for unveiling the mechanism of alkaline tolerance, as well as facilitating the genetic improvement and conservation of Amur ide.

## Results and Discussion

### Transcriptome Sequencing and Assembly of Amur Ide

**Figure 1:**
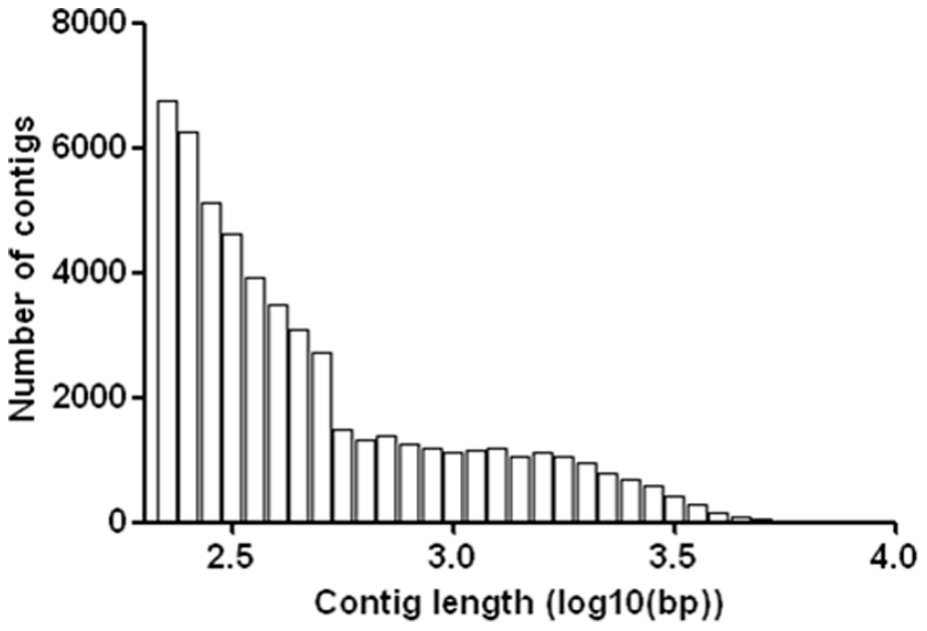
Length distribution of assembled contigs of Amur ide.

To enable a comprehensive understanding and profiling of the transcritpome of Amur ide, mixed RNA originating from 12 tissues was sequenced by using Solexa HiSeq2000 sequencing technology. A total of 99,883,236 paired-end reads were generated with a read length of 101 bp. After the removal of ambiguous nucleotides and low-quality sequences (Phred quality scores <20), a total of 87,740,916 cleaned reads (87.8%) were obtained. The raw transcriptome sequences in this study have been deposited in the NCBI SRA database (Accession number SRR677015). The cleaned reads were then assembled by using Trinity assembler [17]. As shown in Table 1, the trancriptome was assembled, combining 73,668,103 reads into 53,632 contigs, ranging from 107 to 9,691 bp in length. The average length was 647 bp, the N50 length was 1,094 bp and the median length was 356 bp. The contig length distribution is shown in Figure 1.

**Table 1:** Statistics of transcriptome sequencing, assembly and annotation of Amur ide.

Stage		
Sequencing	Number of reads (101-bp paired-end)	99,883,236
	Total bases	9.99 Gb
	Cleaned reads	87,740,916
Assembly	Number of contigs	53,632
	Maximum contig length	9,691 bp
	Minimum contig length	107 bp
	Average contig length	647 bp
	N50 length	1,094 bp
Annotation	Contigs with blast results	30,866
	Unigenes with blast results	19,338
	Contigs with GO terms	13,717
	Unigenes with GO terms	10,674

To validate the assembly, 34 contigs were randomly selected. Primers were designed and transcripts were amplified from a cDNA template of pooled tissues of Amur ide. Thirty-one (91.2%) contigs were successfully amplified with the expected length (Table S1), which confirmed that the assembly using the Trinity algorithm was reliable.

### Functional Annotation

All assembled contigs were first compared with the NCBI non-redundant (nr) protein database for functional annotation by using BLASTx with an e-value cutoff of 1e−10. A total of 30,866 contigs had a significant hit, corresponding to 19,338 unique protein accessions in the nr protein database (Table 2). The gene name of the top BLASTx hit was assigned to each of the contigs with significant hits.

**Table 2:** Summary of BLASTX search results of Amur ide transcriptome.

Database	Amur ide hits	Unique protein	% of total unique proteins
NR	30,866	19,338	
UTR	3,822	2,497	
Refseq/Ensembl			
Zebrafish	31,790	15,759	57.8% of 27,271
Medaka	27,524	13,419	54.4% of 24,661
Tetraodon	27,096	12,952	56.0% of 23,118
Three-spined stickleback	27,996	14,047	50.9% of 27,576

**Figure 2:**
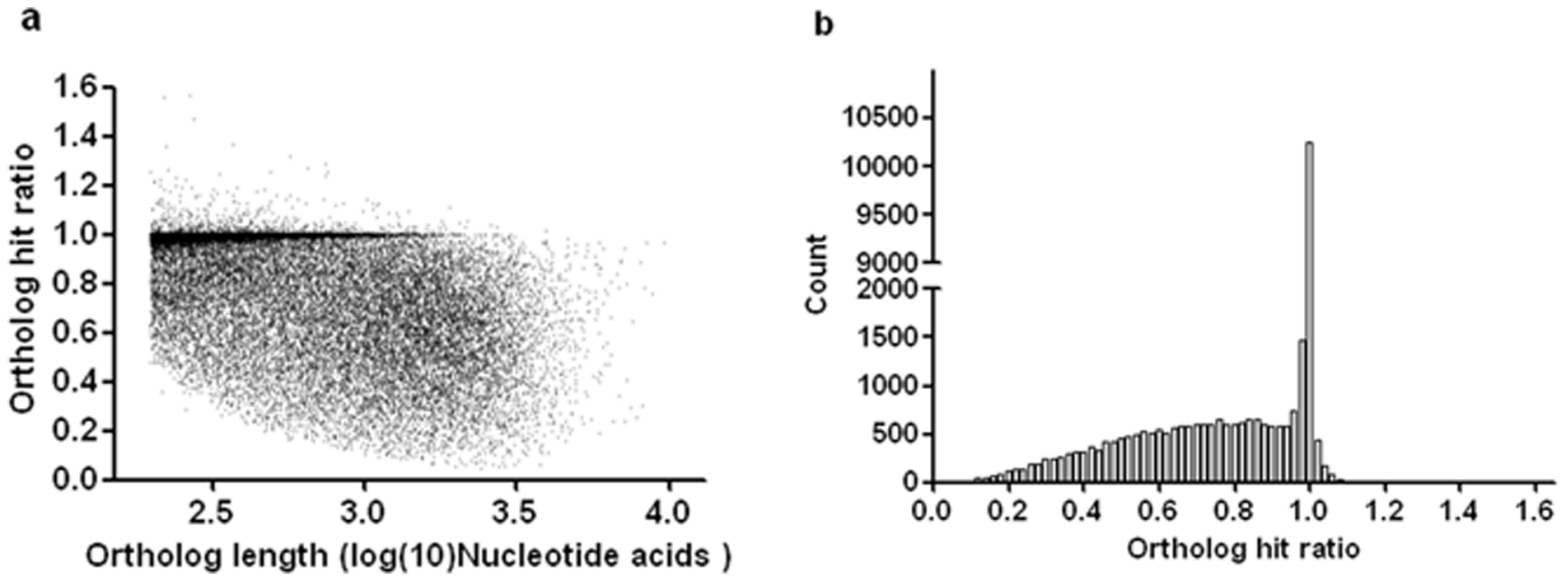
Distribution of ortholog hit ratio and its relationship with ortholog length. Ortholog hit ratios were calculated for contigs with BLASTx results. A ratio of 1.0 indicates the gene is likely fully assembled.

We also calculated the “ortholog hit ratio” [18] by dividing the length of the putative coding region of a unigene by the total length of the ortholog found for that unigene (Figure 2). Each unigene and its best BLASTx hit were considered orthologs and the hit region in the unigene was considered to be a “putative coding region”. Thus, the ortholog hit ratio gives an estimate of the amount of a transcript that is represented by each unigene [19]. Figure 2(a) shows that the completeness of the assembled transcripts only slightly decreases for longer genes, suggesting a high assembly quality in this study, even for long transcripts. The distribution of ortholog hit ratios is represented in Figure 2(b). Most unigenes with BLASTx results had high ortholog hit ratios, indicating a high completeness of these transcripts. Of the 30,866 transcripts with BLASTx results, 14,451 contigs had a ratio ≥0.9 and 26,074 had a ratio ≥0.5. In total only 827 (2.7%) contigs had a ratio greater than 1.0, indicating the possibility of insertions in relative unigenes. The evaluation also suggests that a higher proportion of full length transcripts were in the assembly.

**Figure 3:**
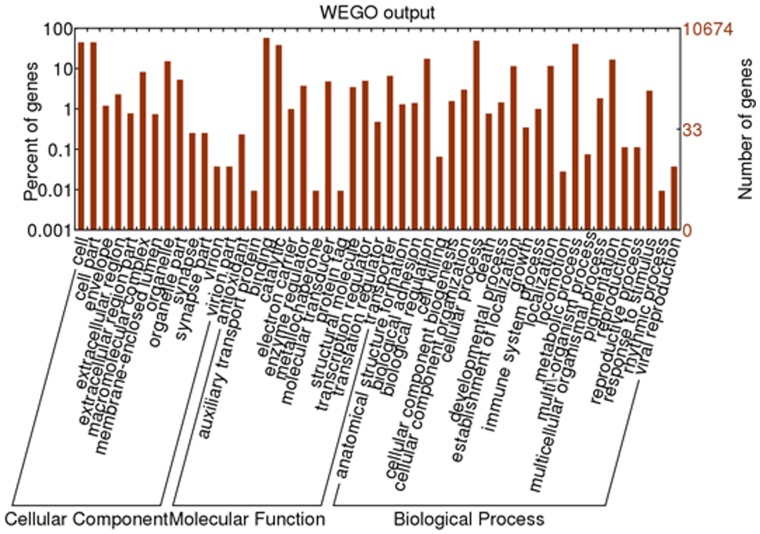
Gene Ontology (GO) categories of the unigenes. Distribution of the GO categories assigned to the Amur ide transcriptome. Unique transcripts (unigenes) were annotated in three categories: cellular components, molecular functions, and biological processes.

Gene ontology (GO) analysis was conducted on those 19,338 unique proteins by using InterProScan (http://www.ebi.ac.uk/Tools/pfa/iprscan/) and integrated protein databases with default parameters. A total of 10,674 unique proteins were assigned at least one GO term for describing biological processes, molecular functions and cellular components. The InterProScan output file was input into the BGI WEGO program and GO annotations were plotted (http://wego.genomics.org.cn) (Figure 3). Of these, the molecular function ontology made up the majority (9,429, 88.3%), followed by the biological process ontology (6,959, 65.2%) and the cellular component ontology (5,029, 47.1%). Briefly, for biological processes, genes involved in cellular processes (GO: 0009987) and metabolic processes (GO: 0008152) were highly represented; for molecular functions, binding (GO: 0005488) was the most represented GO term, followed by catalytic activity (GO: 0003824); cells (GO: 0005623) and organelles (GO: 0043226) were the most represented categories for the cellular component. Interestingly, within biological processes, a total of 303 unigenes were annotated to response to stimulus (GO: 0050896), including 190 unigenes to response to stress (GO: 0006950), 86 unigenes to cellular response to stimulus (GO: 0051716), 50 unigenes to response to external stimulus (GO: 0009605), 35 unigenes to response to chemical stimulus (GO: 0042221), and 4 unigenes to detection of stimulus (GO: 0051606). In previous studies, the GO result of common carp was reported, of which 250 unigenes were annotated to response to stimulus [20]. The expression of more stimulus-related genes in Amur ide from Dali Nor Lake is consistent with the extreme environmental stress of this habitat.

In addition, a KEGG pathway analysis was performed on all assembled contigs as an alternative approach for functional categorization and annotation. Enzyme commission (EC) numbers were assigned to 6,174 unique sequences, which categorized them into different functional groups (Table 3). Briefly, of these sequences with KEGG annotation, 1,975 (32.0%) were classified into metabolism, including major sub-groups of carbohydrate metabolism (826 sequences), lipid metabolism (207 sequences) and amino acid metabolism (188 sequences). Sequences grouped into genetic information processing (GIP), accounted for 1,019 (16.5%), including translation (367 sequences), folding, sorting and degradation (366 sequences), transcription (176 sequences), and replication and repair (110 sequences). Organismal systems, cellular processes and environmental information processing (EIP) groups contained 1,189(19.2%), 836(13.5%) and 538 (8.7%) KEGG-annotated sequences, respectively. A well-categorized and annotated transcriptome could serve as an important and valuable resource for gene identification and functional analysis of alkaline tolerance and osmotic regulation in Amur ide. For instance, 17 transcript contigs associated with osmotic regulation in KEGG analysis have been identified and collected (Table S2).

**Table 3:** KEGG biochemical mappings for Amur ide.

KEGG categories represented	Unique sequences* (Number of KO)
**Metabolism**	**1,975 (1,415)**
Carbohydrate Metabolism	826 (597)
Amino Acid Metabolism	188 (140)
Energy Metabolism	174 (122)
Nucleotide Metabolism	142 (96)
Metabolism of Cofactors and Vitamins	114 (82)
Lipid Metabolism	207 (152)
Glycan Biosynthesis and Metabolism	132 (101)
Metabolism of Other Amino Acids	78 (50)
Xenobiotics Biodegradation and Metabolism	78 (45)
Biosynthesis of Secondary Metabolites	15 (12)
Biosynthesis of Polyketides and Nonribosomal Peptides	21 (18)
**Genetic Information Processing**	**1,019 (812)**
Replication and Repair	110 (88)
Folding, Sorting and Degradation	366 (294)
Transcription	176 (146)
Translation	367 (284)
**Environmental Information Processing**	**538 (398)**
Signal Transduction	378 (275)
Signaling Molecules and Interaction	143 (107)
Membrane Transport	17 (16)
**Cellular Processes**	**836 (600)**
Cell Motility	104 (64)
Cell Growth and Death	178 (141)
Transport and Catabolism	348 (258)
Cell Communication	206 (137)
** ORGANISMAL SYSTEMS**	**1,189 (848)**
Immune System	318 (240)
Endocrine System	202 (149)
Development	80 (48)
Circulatory System	139 (98)
Digestive System	78 (56)
Excretory System	200 (134)
Nervous System	22 (15)
Sensory System	117 (85)
Environmental Adaptation	33 (23)
**Total**	**6,174 (4,073)**

* Unique sequences indicate non-redundant sequences involving particular KEGG category.

**Figure 4:**
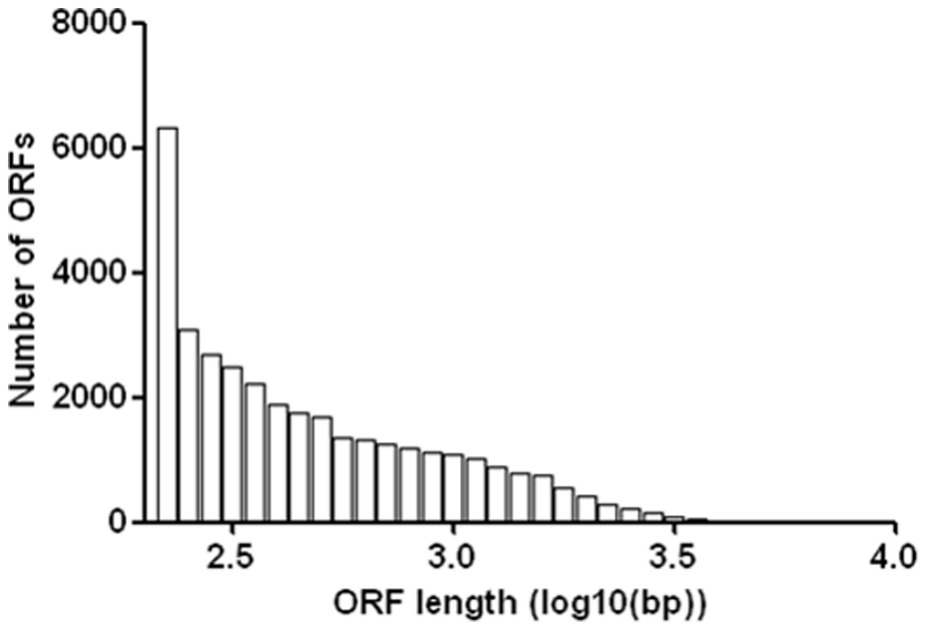
Length distribution of identified ORF.

Of the 53,632 assembled contigs of the Amur ide transcriptome, Open Reading Frames (ORFs) were detected from 34,888 (65.0%) contigs, with an average ORF length of 577 bp and with a range from 51 bp to 9,339 bp (Figure 4). The remaining 18,744 contigs contained no ORFs, indicating they are non-coding sequences and likely come from untranslated regions (UTR). The assembled transcriptome contigs served as a reference for cSNPs identification from RNA-seq data. ORF analysis allows the discrimination of synonymous and non-synonymous SNPs and the identification of nonsense mutations in Amur ide.

### Assessment of Transcriptome Assembly

The assembled contigs of Amur ide transcriptome were compared with Refseq proteins of zebrafish, fugu (*Takifugu rubripes*), medaka *(Oryzias latipes*) and three-spined stickleback (*Gasterosteus aculeatus*) by using the BLASTx program with an e-value cutoff of 1E−10. There were 31,790 contigs (59.3%) with 15,759 unique protein hits, 27,096 contigs (50.5%) with 12,952 unique protein hits, 27,524 contigs (51.3%) with 13,419 unique protein hits and 27,996 contigs (52.2%) with 17,047 unique protein hits identified with significant hits on Refseq proteins of zebrafish, tetraodon, medaka and three-spined stickleback, respectively. The contigs of the Amur ide transcriptome had hits with 50.9% to 57.8% of the unique proteins of zebrafish, tetraodon, medaka and three-spined stickleback (Table 2). Obviously, the closely related zebrafish showed the highest similarity to Amur ide at the gene expression level. However, transcriptome similarity was still relatively lower than expected. Both zebrafish and Amur ide belong to Cyprinidae but are found in different distant subfamilies according to phylogenetic research [21]. It may be that they only share a limited level of similarity. The current research however does not cover the whole transcriptome as the genetic material in this study was only collected from adult fish; transcripts from early development stages were therefore not included. In addition, some rare transcripts may be missed or were only collected as singletons during the assembly, even though a high sequencing depth was applied. For better understanding and characterization of Amur ide transcriptome, we would need a complete set of transcriptome data from virtually every tissue across every life stage and every circumstance, or the whole-genome sequencing and assembly.

### Full-length cDNA Prediction

**Figure 5:**
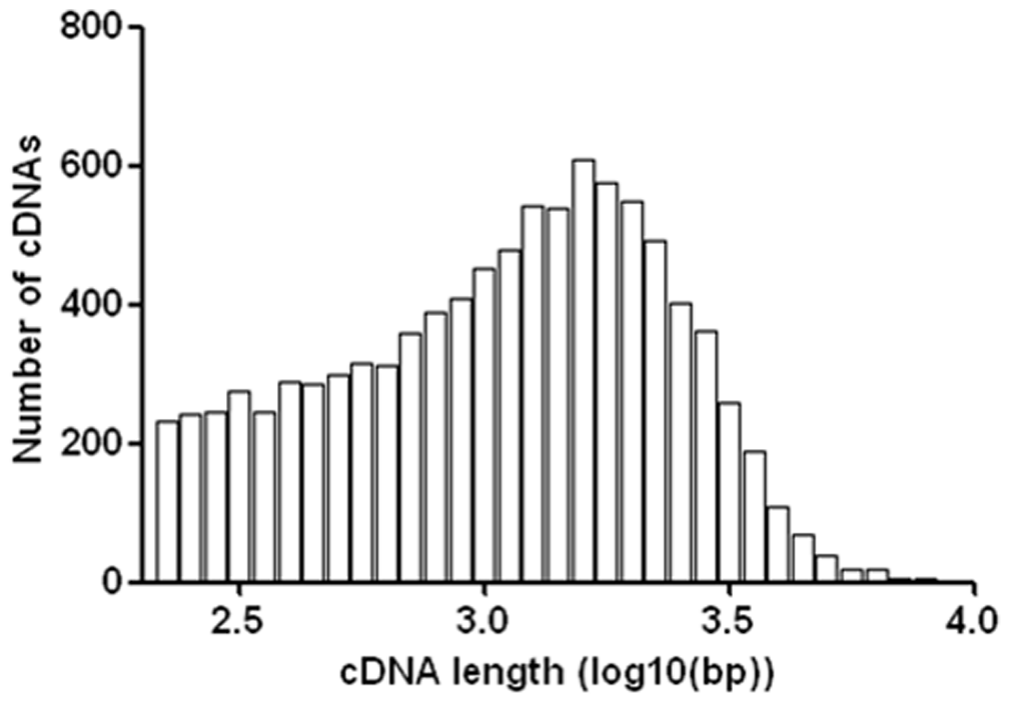
Length distribution of putative full-length cDNAs.

Full-length cDNAs are important resources for many genetic and genomic studies, including gene duplication analysis, alternative splicing and whole genome sequencing and assembly. To identify potential full-length cDNAs with complete ORF in the assembled transcriptome of Amur ide, all contigs were analyzed by the online tool of TargetIdentifier. A total of 9,638 full-length and ORF completely-sequenced sequences were identified from the assembly with a cutoff E-value of 1E−5, with sequence lengths from 201 bp to 8,204 bp (Figure 5). Most of the identified full-length cDNA sequences were shorter than 1.5 kb, suggesting those long full-length cDNA sequences were not easily assembled using only the current set of transcriptome data. The current data may need to be combined with a traditional full-length cDNA library and Sanger’s sequencing method to collect more full-length cDNA sequences and to build a database.

### Repetitive Element Analysis and Microsatellite Identification

A total of 10,395 microsatellites were initially identified from 8,447 contigs, including di-, tri-, tetra-, penta- and hexa-nucleotide repeats. After removing the microsatellites without enough flanking sequences (50 bp) for the design of primers, there were 4,120 unique sequences with microsatellites that can be used to design primers for genotyping (Table 4).

**Table 4:** Statistics of microsatellites identified from Amur ide transcriptome.

Total number of contigs	53,632
Microsatellites identified	10,395
Di-nucleotide repeats	4,316
Tri-nucleotide repeats	749
Tetra-nucleotide repeats	40
Penta-nucleotide repeats	12
Number of contigs containing microsatellites	8,447
Number of microsatellites with sufficient flanking sequences	4,120

The proportion of the repetitive elements in the Amur ide genome was assessed by using Repeatmasker with Vertebrates Repeat Database. Repeatmasking of the 34,750,752 bp of the Amur ide contig sequences resulted in the detection of 890,553 bp (2.56%) of repeated sequences. The classification and respective proportion of the identified repetitive elements are shown in Table S1. The most abundant type of repetitive elements in the sequences were DNA transposons (0.99%), mostly hobo-Activator (0.40%), followed by retroelements (0.61%) including LINEs (0.27%), LTR elements (0.31%), and SINEs (0. 03%). Various satellite sequences, low complexity and simple sequence repeats accounted for 0.10%, 0.48% and 0.33% of the base pairs, respectively.

### SNP Identification

For further application of the RNA-Seq data, SNPs were discovered using the assembled transcriptome. The short reads of RNA-Seq data were aligned onto the reference transcriptome of Amur ide and a total of 79,475,676 (79.6%) reads were mapped on the transcriptome, generating 34,299 SNPs after quality control and filtration (See Methods). The proportions of transition substitutions were 28.5% for A/G and 30.5% for C/T, compared with smaller proportions of transversion for A/C (11.2%), G/T (10.5%), A/T (11.8%) and C/G (7.5%). Among all SNPs detected, 10,408 were in the putative ORF region, of which 4, 335 were synonymous and 6,073 were non-synonymous. The mean number of SNPs per kilobase in the ORF region was 2.64. Further analysis was done to classify identified SNPs (Table 5).

**Table 5:** Classification of SNPs identified from Amur ide transcriptome.

SNP classification	Number of SNPs
5′ UTR	646
3′ UTR	5,159
Coding region	10,408
synonymous	4,335
non-synonymous	6,073
pre-terminated	265
skip-stop-codon	214
mis-sense	5,594
Undefined	18,086
Total	34,299

### Analysis of Synonymous and Non-synonymous Sites

**Figure 6:**
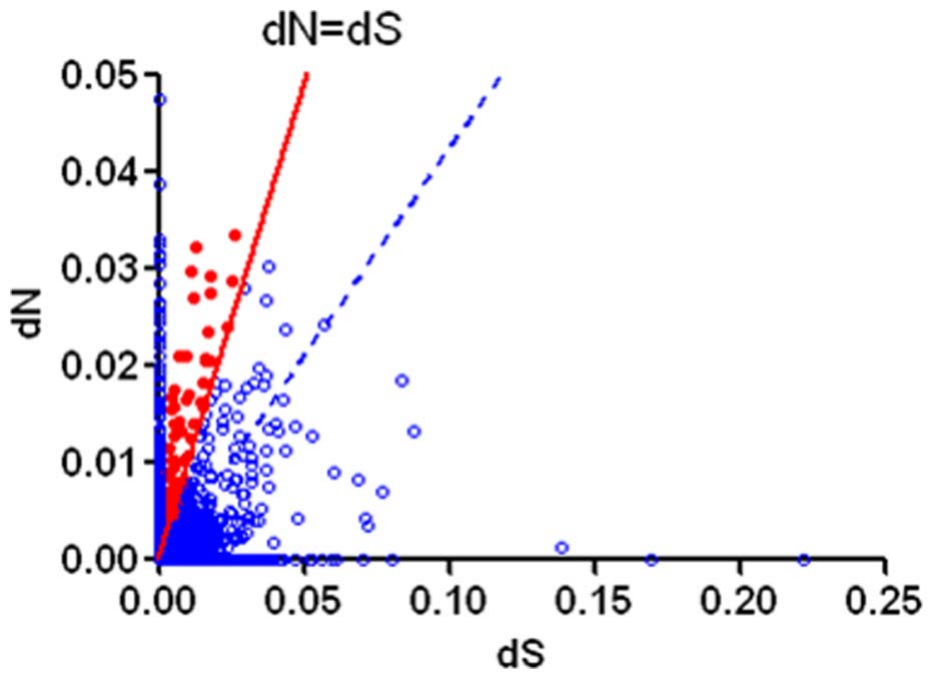
Distribution of SNP non-synonymous (dN) and synonymous (dS) substitution. The solid red line is the null expectation dN = dS. The filled red circles represent unigenes with dN/dS>1. The dashed blue line shows the slope ( = 0.428) of the overall average dN for all contigs/overall average dS for all contigs.

The Amur ide population in Dali Nor Lake has been isolated from other populations for over 10,000 years since the early Holocene. The lake is consistently shrinking, and the alkalinity and salinity are also consistently increasing during this time. How could the Amur ide survive in such an extreme environment? Did the environmental changes speed up gene evolution to adapt to the changing environment? It is commonly known that positive selection likely plays a major role in shaping genetic architecture when populations are placed into new or changing environments [22]–[25]. Thus, we hypothesize that Amur ide experienced strong positive selection on a group of genes and pathways in response to extreme alkalinity and salinity, as well as concentrated heavy metal ions. Non-synonymous (dN) and synonymous (dS) substitution rates have been widely used to measure the intensity of gene evolution [26], [27]. To identify genes undergoing strong positive selection, we estimated dN and dS rates of the assembled genes of Amur ide. A total of 2,646 unigenes which contained at least 1 SNP were used to calculate dN, dS and the dN/dS ratio. The results showed that the overall dN, dS and dN/dS were 0.002, 0.008 and 0.428, respectively, which indicated that most of the genes were not under positive selection. However, the dN/dS ratios of 61 unigenes were greater than 1, indicating strong positive selection did occur on them (Figure 6). The functions of these genes were further investigated either by a pathway analysis or a literature search. Interestingly, a suite of genes were clearly associated with stress adaptation and immunity [28]–[32], including carbonic anhydrase 4, superoxide dismutase, and glutathione S-transferase A (Table 6 and Table S3).

**Table 6:** Unigenes showing positive selection (dN/dS>1) corresponding to stress adaption or immune response.

Unigenes	Uniprot ID	Description	dN/dS
contig7468	sp|P20702|ITAX_HUMAN	Integrin alpha-X	4.18
contig256	sp|P11364|TCB_FLV	T-cell receptor beta chain T17T-22	3.57
contig9830	sp|Q95118|IL2RG_BOVIN	Cytokine receptor common subunit gamma	3.46
contig344	sp|P49616|UPAR_RAT	Urokinase plasminogen activator surface receptor	3.30
contig1256	sp|P40189|IL6RB_HUMAN	Interleukin-6 receptor subunit beta	2.78
contig3025	sp|P11911|CD79A_MOUSE	B-cell antigen receptor complex-associated protein alpha chain	2.64
contig1148	sp|P48284|CAH4_RAT	Carbonic anhydrase 4	2.59
contig3210	sp|P08317|IL8_CHICK	Interleukin-8	2.59
contig6695	sp|P08294|SODE_HUMAN	Extracellular superoxide dismutase [Cu-Zn]	2.30
contig11187	sp|P01873|MUCM_MOUSE	Ig mu chain C region membrane-bound form	2.22
contig12382	sp|Q9NVE5|UBP40_HUMAN	Ubiquitin carboxyl-terminal hydrolase 40	1.96
contig13125	sp|P13387|EGFR_CHICK	Epidermal growth factor receptor	1.86
contig21350	sp|Q66S61|MBL2_CALJA	Mannose-binding protein C	1.77
contig941	sp|P10820|PERF_MOUSE	Perforin-1	1.74
contig59	sp|P06314|KV404_HUMAN	Ig kappa chain V-IV region B17	1.60
contig21332	sp|A7M9B2|YCF1_CUSRE	Putative membrane protein ycf1	1.55
contig21463	sp|Q6PIU2|NCEH1_HUMAN	Neutral cholesterol ester hydrolase 1	1.54
contig421	sp|P04114|APOB_HUMAN	Apolipoprotein B-100	1.54
contig11654	sp|P20759|IGHG1_RAT	Ig gamma-1 chain C region	1.41
contig255	sp|Q28085|CFAH_BOVIN	Complement factor H	1.32
contig895	sp|P30568|GSTA_PLEPL	Glutathione S-transferase A	1.31
contig2382	sp|Q8BK26|FBX44_MOUSE	F-box only protein 44	1.31
contig15120	sp|Q91009|NTRK1_CHICK	High affinity nerve growth factor receptor	1.27
contig7962	sp|Q9MZV7|CASP1_CANFA	Caspase-1	1.25
contig1236	sp|O95415|BRI3_HUMAN	Brain protein I3	1.18
contig10013	sp|Q80SU7|GVIN1_MOUSE	Interferon-induced very large GTPase 1	1.17
contig1492	sp|P19181|HV05_CARAU	Ig heavy chain V region 5A	1.11
contig1317	sp|P15684|AMPN_RAT	Aminopeptidase N	1.06
contig11745	sp|Q96G23|CERS2_HUMAN	Ceramide synthase 2	1.02
contig13871	sp|P29533|VCAM1_MOUSE	Vascular cell adhesion protein 1	1.02
contig3775	sp|P50283|CD7_MOUSE	T-cell antigen CD7	1.01
contig17067	sp|Q16787|LAMA3_HUMAN	Laminin subunit alpha-3	1.01

We looked into several typical genes which respond to stress. Carbonic anhydrase (CA) is a zinc metalloenzyme that catalyzes the hydration of CO_2_ to provide H^+^ and HCO_3_^−^ for ion transport processes. CA is widely recognized as a response to environmental stress in eukaryotes; for instance, CA was induced in the gills of the euryhaline green crab, *Carcinus maenas*, when the crab was transferred between different salinities [33]; Increasing salinities or an alkaline shift could induce CA in green alga *Dunaliella salina* [34]; CA even plays a role in cold adaptation among Antarctic fish [35]. Glutathione S-transferases (GSTs) are a large family of detoxification enzymes contributing to the biotransformation of a wide variety of environmental xenobiotics. They are thought to play major roles in oxidative stress, being responsible for various stress tolerance in plants [36]–[38] and animals [39]–[41]. GST from wild soybean (*Glycine soja*) was transferred into tobacco, enhancing drought and salt tolerance in transgenic tobacco [42]. GST is even used as a stress biomarker in mollusc species to monitor fuel oil spills [43].

Superoxide dismutases (SOD) are enzymes that catalyze the disputation of superoxide into oxygen and hydrogen peroxide and are well known to respond to various environmental stresses in eukaryotes. Overexpressing chloroplastic Cu/Zn SOD in plants may increase resistance to oxidative stress [44]. In animals, high levels of Cu/Zn SOD were detected in spermatogonia, protecting from oxidative stress [45]. Similar to GST, SODs are also used as an important biomarker in aquatic organisms for monitoring toxic environmental pollutants [46], [47].

The results of the dN/dS analysis revealed fast genome evolution in some genes probably in order to adapt the extreme environmental stress in Dali Nor Lake and confirmed our hypothesis that increasing salinity, alkalinity and heavy metal concentration likely resulted in powerful selective pressures on certain genes for new genotypes that were better suited these stressful conditions.

### Conclusions

In this study, the transcriptome of Amur ide was sequenced using the HiSeq2000 platform with high coverage, and then *de novo* assembled and functionally annotated by comparing with exiting protein databases of closely related species. An ORF analysis was conducted and a large number of full length cDNA sequences have been identified. In addition, repetitive element analysis was conducted, and cDNA SSR and SNP loci were identified for future marker development and genetic analysis. Synonymous and non-synonymous sites were analyzed on unigenes, which revealed that the Amur ide population in Dali Nor Lake has experienced fast evolution to adapt the extreme alkaline-saline environment.

## Methods

### Ethics Statement

This study was approved by the Animal Care and Use committee of the Centre for Applied Aquatic Genomics at Chinese Academy of Fishery Sciences.

### Biological Samples

Ten wild Amur ide were sampled at the north shore of Dali Nor Lake, Inner Mongolia, China on May 12, 2012. Twelve tissues, including brain, muscle, liver, intestine, blood, head kidney, trunk kidney, skin, gill, spleen, gonad and heart, were dissected and collected. Tissue samples were stored in RNAlater (Qiagen, Hilden, Germany), transported to the laboratory at room temperature, and then stored at −20°C prior to RNA extraction.

### RNA Extraction

Total RNA was extracted from 12 tissues using the TRIZOL Kit (Invitrogen, Carlsbad, CA, USA) following manufacturer’s instructions. RNA samples were then digested by DNase I to remove potential genomic DNA. Integrity and size distribution were checked with Bioanalyzer 2100 (Agilent technologies, Santa Clara, CA, USA). Equal amounts of the high quality RNA samples from each tissue were then pooled for cDNA synthesis and sequencing.

### cDNA Library Construction, Sequencing and Assembly

RNA-seq library preparation and sequencing was carried out by HudsonAlpha Genomic Services Lab (Huntsville, AL, USA) as previously described [13]. cDNA libraries were prepared with ∼2.5 µg of starting total RNA following the protocols of the Illumina TruSeq RNA Sample Preparation Kit (Illumina). The final library had an average fragment size of ∼270 bp and final yields of ∼400 ng. After KAPA quantitation and dilution, the library was sequenced on an Illumina HiSeq 2000 with 101 bp paired-end reads. All sequenced reads in SRA format have been uploaded to the NCBI Short Read Archive with the accession number of SRR677015. Adaptor sequences were trimmed and reads with low quality or length less than 10 were further removed by SolexaQA software [48]. Cleaned reads were used for *de novo* assembly by TRINITY with default parameters.

### Functional Annotation

The assembled transcriptome contigs were subjected to a similarity search against NCBI non-redundant (nr) protein database using BLASTx with an e-value cutoff of 1E−10. Gene names and descriptions were assigned to each contig based on the top BLASTx hit with the highest score. Gene ontology (GO) analysis was then conducted on the assembled transcriptome by using InterProScan (http://www.ebi.ac.uk/Tools/pfa/iprscan/) and integrated protein databases with the default parameters. The GO terms associated with transcriptome contigs were then obtained to describe the genes in the areas of biological processes, molecular functions and cellular components. The InterProScan output file was input into BGI WEGO program and the GO annotations were plotted (http://wego.genomics.org.cn). All assembled contigs were analyzed by ESTScan to search for ORFs, which could distinguish between coding and non-coding sequences.

KEGG pathways were assigned to assembled contigs using the online KEGG Automatic Annotation Server (KAAS) (http://www.genome.jp/tools/kaas/) [49]. The Bi-directional Best Hit (BBH) method was used to obtain KEGG Orthology (KO) assignment.

### Assembly Assessment

To compare the similarity to other teleost species, the transcriptome contigs were compared to Refseq and Ensemble proteins of zebrafish, fugu (*Takifugu rubripes*), medaka *(Oryzias latipes*) and three-spined stickleback (*Gasterosteus aculeatus*), as well as the transcriptome of Amur ide by using the BLAST program with default parameters.

### Full-length cDNA Identification

Putative full-length cDNAs were identified by using the online tool TargetIdentifier [6], [50] and comparing to non-redundant protein databases with a cutoff e-value of 10^−5^. The cDNA sequence was recognized as a full-length cDNA only if the start codon (ATG) and poly (A) tail were identified.

### Repetitive Element Analysis and Microsatellite Identification

To identify all repetitive elements in the assembled transcriptome, RepeatMasker was used with Repbase for all vertebrates and zebrafish. A perl-based script Msatfinder V 2.0.9 [51] was used for microsatellite identification from assembled cDNA contigs. The mononucleotide repeats were ignored by modifying the configure file. The repeat thresholds for di-, tri-, tetra-, penta-, hexa-nucleotide motifs were set as 8, 5, 5, 5 and 5 respectively. Only microsatellite sequences with flanking sequences longer than 50 bp on both sides were identified for future marker development.

### SNP Identification and dN/dS Analysis

To identify putative single nucleotide polymorphism (SNP) loci in the transcriptome of Amur ide, all RNA-Seq reads were mapped onto the assembled transcriptome using BWA and SAMtools. The filtering threshold was set as bellowing, the read depth to no less than 10, and the quality score to no less than 20. dN and dS were calculated by KaKs_Caculator 1.2 [52], [53]. An input file (*.axt) for the KaKs_Caculator was generated from the ORF sequence and SNP list, which contains ID, reference sequence and sequence with SNPs for each gene. Then the output file was further extracted for useful information.

## Supporting Information

Table S1 **Validation of assembled contigs by PCR.**
(XLS)

Table S2 **Osmotic regulation related genes by KEGG analysis.**
(XLS)

Table S3 **All 61 unigenes putatively under positive selection.**
(XLS)
